# Dissociation of Bone Resorption and Bone Formation in Adult Mice with a Non-Functional V-ATPase in Osteoclasts Leads to Increased Bone Strength

**DOI:** 10.1371/journal.pone.0027482

**Published:** 2011-11-07

**Authors:** Kim Henriksen, Carmen Flores, Jesper S. Thomsen, Anne-Marie Brüel, Christian S. Thudium, Anita V. Neutzsky-Wulff, Geerling E. J. Langenbach, Natalie Sims, Maria Askmyr, Thomas J. Martin, Vincent Everts, Morten A. Karsdal, Johan Richter

**Affiliations:** 1 Nordic Bioscience A/S, Herlev, Denmark; 2 Molecular Medicine and Gene Therapy, Lund University, Lund, Sweden; 3 Institute of Anatomy, University of Aarhus, Aarhus, Denmark; 4 Department of Functional Anatomy, Academic Centre of Dentistry Amsterdam (ACTA), University of Amsterdam and VU University Amsterdam, Research Institute MOVE, Amsterdam, The Netherlands; 5 St. Vincent's Institute for Medical Research, Melbourne, Australia; 6 Department of Oral Cell Biology, Academic Centre of Dentistry Amsterdam (ACTA), University of Amsterdam and VU University Amsterdam Research Institute MOVE, Amsterdam, The Netherlands; Florida International University, United States of America

## Abstract

Osteopetrosis caused by defective acid secretion by the osteoclast, is characterized by defective bone resorption, increased osteoclast numbers, while bone formation is normal or increased. In contrast the bones are of poor quality, despite this uncoupling of formation from resorption.

To shed light on the effect of uncoupling in adult mice with respect to bone strength, we transplanted irradiated three-month old normal mice with hematopoietic stem cells from control or *oc/oc* mice, which have defective acid secretion, and followed them for 12 to 28 weeks.

Engraftment levels were assessed by flow cytometry of peripheral blood. Serum samples were collected every six weeks for measurement of bone turnover markers. At termination bones were collected for µCT and mechanical testing.

An engraftment level of 98% was obtained. From week 6 until termination bone resorption was significantly reduced, while the osteoclast number was increased when comparing *oc/oc* to controls. Bone formation was elevated at week 6, normalized at week 12, and reduced onwards. µCT and mechanical analyses of femurs and vertebrae showed increased bone volume and bone strength of cortical and trabecular bone.

In conclusion, these data show that attenuation of acid secretion in adult mice leads to uncoupling and improves bone strength.

## Introduction

Bone remodeling is a continuous process that maintains calcium homeostasis, removes old bone and mediates microfracture repair, thereby ensuring bone quality [1]. Bone resorption is performed by osteoclasts, after which the osteoblasts form new bone matrix, leading to restoration of the removed bone [2]. These two processes are normally tightly balanced, a process referred to as coupling [3], . Recent studies have indicated that the coupling of bone formation to bone resorption is more complex than originally thought [5], [ 6], and likely includes secretion of bone anabolic factors by the osteoclasts, independent of bone resorptive activity [2], [ 7].

Osteoclasts derive from hematopoietic stem cells which, in the presence of the osteoblast-derived molecules RANKL and M-CSF, develop into mature multinucleated bone resorbing osteoclasts [8], [ 9]. The osteoclasts resorb bone by secretion of hydrochloric acid and proteases which, in combination, dissolve the calcified bone matrix [8], [ 9]. Acidification of the resorption compartment is achieved by active proton transport mediated by the osteoclast specific V-ATPase, while chloride is secreted by the chloride-proton antiporter ClC-7 [10]–[14].

Loss of function mutations or gene knockouts in humans and mice of these two molecules lead to different types of osteopetrosis indicating their importance for dissolution of the inorganic bone matrix [10], [ 15], [ 16]. These forms of osteopetrosis are characterized by normal or even increased indices of bone formation despite the presence of high numbers of non-resorbing osteoclasts [17]-[20], indicating that bone resorption and bone formation are no longer coupled. Despite the high bone mass, a feature of osteopetrosis is poor bone quality, which has been speculated to be due to the extreme suppression of bone resorption [21], [ 22], the failure to resorb calcified cartilage [9], and to hyper-activity of the osteoblasts [23].

A recent study of ClC-7 deficient mice indicated uncoupling of bone formation from bone resorption [24]. However, further characterization failed to confirm these findings due to the severe developmental phenotype, where calcified cartilage completely occluded the marrow cavity of all long bones [25]. This illustrates the difficulty of investigating bone phenotypes in these very young mice.

The *oc/oc* mice exhibit very severe osteopetrosis due to a mutation in the a3 subunit of the V-ATPase, and these mice die of anemia 3–4 weeks after birth [26]. Recent studies in these mice have shown that the osteopetrotic phenotype can be rescued by neonatal transplantation of normal or gene-corrected hematopoietic stem cells into irradiated mice, in accordance with the hematopoietic nature of the defect [27]–[30].

In order to investigate the effect of osteopetrosis on bone quality in adult mice and also shed light on the uncoupling observed in some forms of osteopetrosis, we induced osteopetrosis in normal 3- month old mice by transplanting them with fetal liver derived hematopoietic stem cells from *oc/oc* mice or their corresponding control littermates, and then followed them for three or six months and characterized their bone and osteoclast phenotypes in detail.

## Materials and Methods

### Mice

Breeding pairs of (C57BL/6J _ C3HheB/FeJ) F1 oc/+ mice (CD45.2) were obtained from the Jackson Laboratory (Bar Harbor, ME) and maintained in the conventional animal facility at the Biomedical Centre, University of Lund.

All experiments were performed according to protocols approved by the local animal ethics committee in both Denmark (Rådet for Dyreforsøg (The Animal Experiments expectorate)) registration number 2007/561-1303 and Sweden (Malmö/Lunds Djurförsöksetiske Nämnd (The ethics committee for animal studies in Malmö/Lund) registration number M 128-09.

### Genotyping of mice

Mice were genotyped on the day of birth using DNA extracted from the tip of the tail as described previously [27].

### Harvest and isolation of fetal liver hematopoietic cells

On embryonic day 14.5, pregnant mice were killed by CO_2_ poisoning, and embryos were removed. Fetal livers (FLs) were dissected out and put into PBS (Invitrogen) supplemented with 2% FCS (Invitrogen). Single-cell suspensions were prepared by drawing liver cells through a 23-gauge needle followed by filtering through a 50 µm cell strainer. Individual FLs were genotyped by lysing a cell sample and running the PCR described above. Cells from both wild type (+/+) and *oc*/+ embryos were used as controls and henceforth designated as such, as oc/+ mice are phenotypically indistinguishable from +/+ littermates.

### Transplantation and follow-up

Three-month-old mice (C57BL/6J _ C3HheB/FeJ)(CD45.1) were irradiated with 950 cGy administered from a 137Cs source. Four hours later mice received an intravenous transplant of 2×10^6^ freshly thawed FL cells in 300 µL PBS. To avoid infection following transplantation the animals were treated for 14 days with Baytril in their drinking water. After transplantation the two groups of mice were followed for 3 months. Intraperitonal injections of calcein (20mg/kg) were given 10 and 3 days prior to sacrifice.

For the 12 week experiment a total of 10 mice were transplanted, 5 controls and 5 *oc/oc*, and for the 28 week experiment a total of 11 mice were transplanted, 5 controls and 6 *oc/oc*. Of all the mice 1 control died of the 12 week and 2 controls died of the 28 week experiment, excluding these from the analyses. The deaths did not appear to be related to the transplantation procedure.

At termination the bones for µCT and mechanical testing in the 12 week experiment were stored in Lilly's fluid until analysis after which they were transferred to 0.9% NaCl and 0.1% NaN_3_, while the bones from the 28 week experiment we stored in 0.9% NaCl and 0.1% NaN_3_ at all time points. A published study clearly showed that fixation does not impact measurements of bone strength (*F*_max_) in mice [31], and thus all samples were treated equally in the mechanical test (see later).

### Engraftment and lineage distribution analysis of peripheral blood

Peripheral blood (PB) was collected in heparin (LEO Pharma, Thornhill, ON) after tail clipping of mice, and mixed with equal volumes of PBS containing 2% FCS. Following centrifugation, the supernatant was poured off, erythrocytes were lysed with NH_4_Cl, and the cells were washed twice with PBS containing 2% FCS. Subsequently, cells were incubated on ice for 20 to 30 minutes with APC-conjugated antibodies directed against B220, CD3, Gr-1, and Mac-1 multilineage analysis) (Becton Dickinson). The cells were suspended in 300 µL PBS containing 2% FCS followed by addition of 1 µg/mL 7-amino-actinomycin D (7-AAD, for detection of nonviable cells; Sigma, St Louis, MO) before analysis using a fluorescence-activated cell sorting (FACS) Calibur Instrument (Becton Dickinson).

### Serum collection

All sera were collected by retro-orbital bleeding after overnight fasting of the mice 6, 12, 18, 23 and 28 weeks after transplantation.

### Bone Resorption by Mature Osteoclasts

Isolated spleen cells from either genotype were differentiated into mature osteoclasts by 4 days of culture in αMEM + M-CSF (25 ng/mL), trypsinization, and reseeding at 900,000 cells/six-well plate, followed by 7 days of culture in αMEM containing RANKL (100 ng/ml) and M-CSF (25 ng/ml) with media exchanged every day as described by Neutzsky-Wulff et al. [39]. Mature osteoclasts from either transplantation group were lifted using trypsin and cell scraping and reseeded on cortical bone slices (see reference [39]), at 50,000 cells/bone slice. Culture supernatants were collected and stored at -20°C until further analysis.

### Bovine cortical bone slices

Bovine cortical bone from cows of more than 3 years of age was cut into thin slices (0.5 cm diameter) as described by Neutzsky-Wulff et al. [39] and stored in 70% ethanol until use. Prior to seeding of cells, bone slices were washed thoroughly in the appropriate medium.

### Measurement of TRAP Activity in Cell Culture Supernatants

TRAP activity in cell culture medium was measured as described previously [32]. Briefly, samples were incubated with TRAP reaction buffer, containing p-nitrophenyl phosphate and sodium tartrate, for 1 hour at 37°C in the dark. The reaction was stopped with 0.3 M NaOH. Absorbance was measured in an ELISA reader at 405 nm with 650 nm as reference.

### Biochemical Markers of Bone Turnover in serum

TRAP5b activity in serum was measured by the Mouse-TRAP assay (SD-TR103, IDS) according to the manufacturer's protocol. Serum samples from individual mice were diluted in PBS to obtain readings within the range of the kit.

Alkaline phosphatase (ALP) was measured by mixing serum samples or controls with substrate solution (0.95 ml AMP buffer [50 ml Milli Q water, 6.25 ml 2-amino-2-methyl-1-propanol 95% {A65182, Sigma}, pH adjusted to 10.0, volume adjusted to 62.5 ml by addition of Milli Q water], 9.5 ml Milli Q water, 40 mg PNPP [P5994, Sigma], 190 µL 1M MgCl_2_) and incubating for 20 minutes in the dark. The reaction was stopped by addition of 0.5 M NaOH. Colorimetric changes were measured at 405 nm with 650 nm as reference using an ELISA reader.

C-terminal type I collagen fragments (CTX-I) were measured using the RatLaps ELISA (1RTL4000; IDS Nordic A/S, Herlev, Denmark), according to the manufacturer's protocol.

Serum P1NP was measured using an ELISA (IDS Nordic A/S, Cat#AC-33F1) according to the manufacturer's instructions.

### Micro-computed tomography (micro-CT) imaging

Three-dimensional reconstructions of trabecular and cortical bone of the lumbar vertebrae and femurs were generated with a high-resolution micro-CT system (µCT 40; Scanco Medical AG, Brüttisellen, Switzerland). The bones were mounted in a cylindrical specimen holder to be captured in a single scan. They were secured with synthetic foam and were completely submerged in physiological saltwater containing 0.1% NaN_3_. Scans with an isotropic resolution of 10 µm were made using a 55-kV peak-voltage X-ray beam. Each scan projection (300 ms) was performed four times and averaged to optimize the signal-to-noise ratio, thereby facilitating segmentation. The computed linear attenuation coefficient of the X-ray beam in each volume element (voxel) was stored in an attenuation map and represented by a gray value in the reconstruction. Specific volumes of interest (VOIs) were selected. The complete vertebral trabecular bone was selected for analysis. To analyze the femur trabecular bone, a region of 5% of the bone length distal of the metaphysis was evaluated. Cortical bone analysis was performed in the region between 45 and 55% along the length of the femurs. To discriminate between bone and background, the reconstructions were segmented using an appropriate fixed threshold. For cortical and trabecular bone this threshold was the grey value comparable to respectively 500 and 350 mg hydroxyapatite/cm^3^. Multiple cortical and trabecular bone parameters were determined using morphometric software supplied by the manufacturer [for trabecular bone: bone volume fraction (BV/TV), trabecular thickness (Tb.Th), and degree of mineralization of the bone (DMB); for cortical bone: Cortical bone volume (Ct.BV), cortical thickness (Ct.Th), degree of mineralization of the bone (DMB), endocortical diameter (Ec.Dm), endocortical marrow volume (Ec.M.Vol), and periosteal diameter (P.Dm)].

### Bone Strength Measurements

#### Femoral Diaphysis

The femora were carefully cleaned from muscles and soft connective tissue. The length of the left femora was measured using an electronic caliper and the mid-point of the femora was marked with a permanent marker pen. The femora were placed in a testing jig for three-points bending with their posterior surface resting on two lower supports located 6.6 mm apart, with their midpoint centered between the two lower supports. The testing jig was then placed in an Instron materials testing machine (model 5566, High Wycombe, UK) and load was applied at a constant deformation rate of 2 mm/min with a rod at the upper anterior midpoint of the femur. During compression testing load-deformation data were recorded using Merlin (version 3.21, Instron, High Wycombe, UK), stored on an attached PC for later analysis. After testing, the fracture line was examined to ensure the fracture occurred perpendicular to the longitudinal axis of the bone. Maximum load (*F*_max_, N) was determined from the load-deformation data using in-house developed software.

#### Femoral Neck

The proximal femur (the proximal half obtained after the three-point bending test) was mounted in a custom-made device for standardized fixation [33]. The fixation device holding the specimen was then placed into the material testing machine, and a vertical load exerted by a cylinder was applied to the top of the femoral head. The cylinder was directed parallel to the axis of the femoral diaphysis and moved at a constant rate of 2 mm/min until fracture of the femoral neck. During biomechanical testing, load-deformation values were obtained and stored on the PC for later analysis. Maximum load (*F*_max_, N) was determined from the load-displacement data using in-house developed software.

#### Vertebral Body

The fourth lumbar vertebral body was dissected free from L3 and L5 and the posterior processes were carefully removed under a dissecting microscope using a fine electric saw and a small clipper.

The cartilaginous endplates were removed with a small scalpel in a fashion that left parallel planes at the cranial and caudal ends without removing excess bone, resulting in a bone specimen height of approximately 2.8 mm. The vertebral bone specimens were placed in the materials testing machine between two parallel plates and compression tested at a constant velocity of 2 mm/min until failure. During biomechanical testing, load-deformation values were obtained and stored on the PC for later analysis. Maximum load (*F*_max_, N) was determined from the load-deformation data using in-house developed software.

### Histomorphometry and staining of plastic embedded specimens

For specimens destined for plastic embedding, the hind legs were fixed in 3.7% formaldehyde in PBS and stored in 70% ethanol. Tibias were embedded in methylmethacrylate in a fully calcified state as previously described [34]. Sections of 5 µm thickness were cut, and stained with each of the following solutions: Toluidine blue, Safranin O/fast green, Goldner's trichrome, Xylenol Orange (counterstain for calcein labeled specimens) and TRACP stain. Histomorphometry was carried out according to standard procedures [35] in the proximal tibia using the Osteomeasure system (OsteoMetrics Inc.). Standard histomorphometric measurements were performed on toluidine blue stained sections in a region 1.1 mm long commencing 370 µm from the end of the hypertrophic zone of the growth plate. Calculations of mineral apposition rate (MAR) were based only on measurements of doubled labeled surfaces (dLS), which were measured in the same region.

### Assessment of bone structure by histology

Humeri were decalcified in 15% EDTA and embedded in paraffin. Cutting was done on a HM360 microtome (Micron) at a 5 µm thickness. The sections were stained with hematoxylin and observed through an Olympus BX60 microscope using a 20x/0,40 objective polarized through filters U-ANT and U-POT. Images were obtained with a DP71 digital camera (Olympus) using the Cel&&A software (Olympus).

### Statistics

All statistical calculations were performed by Student's two-tailed unpaired *t*-test, assuming normal distribution and equal variance, with a significance level of P<0.05 (NS: not significant; *:p<0.05, **:p<0.01, ***:p<0.001). Error bars indicate standard error of the mean (SEM).

## Results

### Experimental setup and engraftment analysis

**Figure 1:**
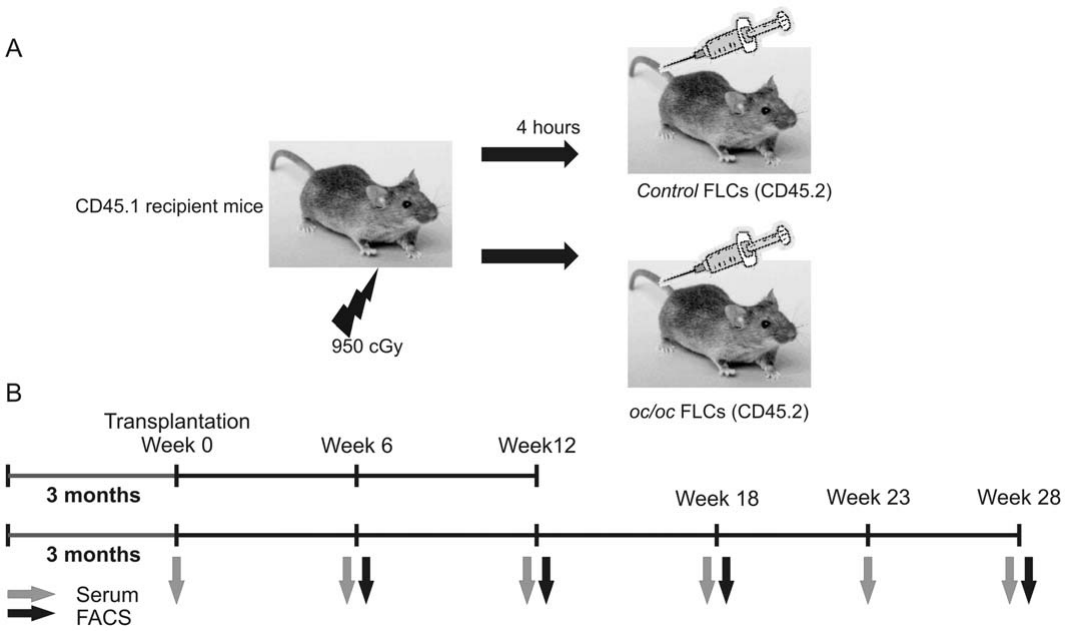
Schematic illustration of the experimental design. A) Illustration of the irradiation and transplantation setup. B) Overview of the timeline and sample collection times from the 12 week and 28 week experiments.

Figure 1 shows the experimental setups. No signs of hepatosplenomegaly were observed in any of the experiments (data not shown).

**Figure 2:**
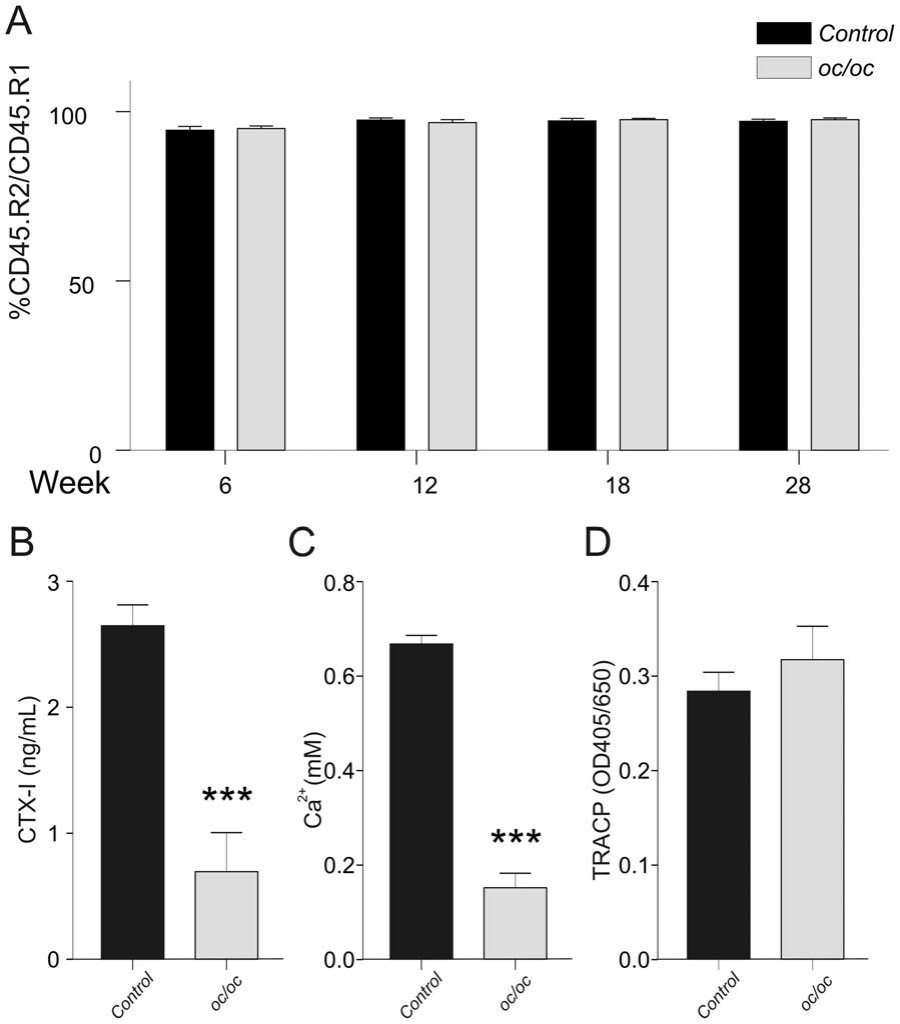
Engraftment analysis and *in vitro* bone resorption. A) Flow cytometry analysis of peripheral blood samples stained with an antibody against CD45.2 to quantify the level of engraftment. Flow cytometry was conducted in samples from all mice (see Methods section) and at the time points indicated. B-D) Splenocytes were isolated and cultured on bovine cortical bone in the presence of RANKL and M-CSF. At day 10 bone resorption was measured by CTX-I (B) and calcium release (C) release and osteoclast numbers measured by TRACP activity in the supernatants (D). Osteoclast cultures are representative of two individual experiments with 6 replicates of each condition.

At week 6 the ratio of CD45.2 (donor) cells to CD45.1 (host) cells in peripheral blood was approximately 95% in both groups (Figure 2A), and at 12, 18, and 28 weeks an engraftment level of approximately 98% was obtained in both groups, confirming successful transplantation. Since *oc/oc* mice have altered cellular composition of the hematopoietic compartment [36], an analysis of the major hematopoietic lineage cells was conducted. This showed no changes in the levels of B220+, CD3+, Mac1_High_/Gr1_low_, and Mac1_low_/Gr1_high_ cell populations between the two groups (Figure S1).

At termination splenocytes and bone marrow cells were isolated and cultured on cortical bone slices for 10 days to investigate osteoclastogenesis and function. As seen in figure 2B-C bone resorption measured by calcium release and CTX-I is significantly reduced in spleen-derived osteoclasts from mice transplanted with *oc/oc cells* when compared to osteoclasts derived from control animals. Furthermore, measurements of the osteoclast marker TRACP activity in the supernatants showed no changes in osteoclast numbers, as seen *in vitro* for both ClC-7 and *Atp6i* deficient mice (Figure 2D)[24]; [37]. Similar data were obtained with bone marrow derived osteoclasts (data not shown).

### Assessment of bone volume

**Figure 3:**
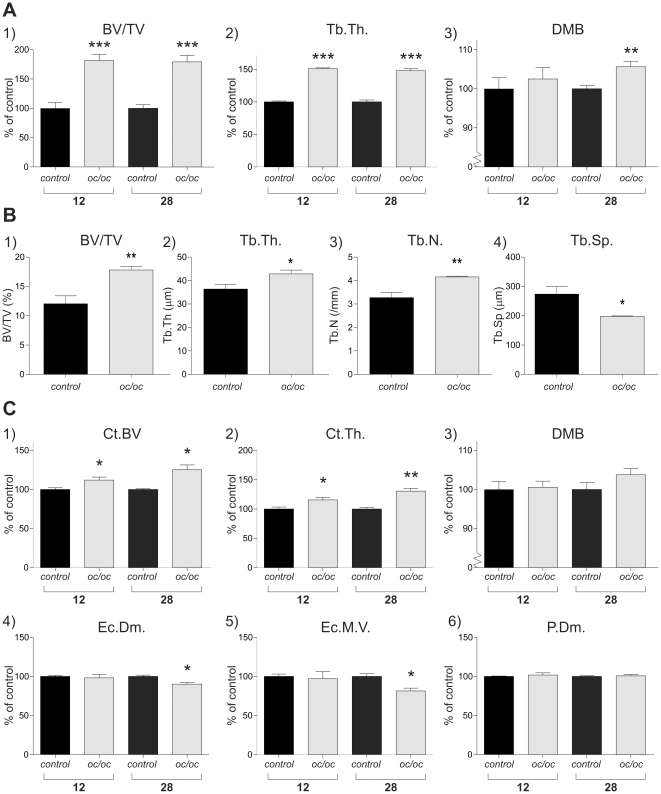
Assessment of bone volume. A) µCT analysis of the vertebrae from both the 12 and the 28-week experiment. For comparison the control group was normalized to 100%. 1) Bone volume/Total Volume (BV/TV) in % of control, 2) Trabecular Thickness (Tb.Th.) in % of control, and 3) Degree of Mineralization of the Bone (DMB) in % of control. B) Bone histomorphometry on vertebrae from the 12-week experiment. 1) Bone volume/Total Volume (BV/TV), 2) Trabecular Thickness (Tb.Th.), 3) Trabecular Number (Tb.N.), and 4) Trabecular Spacing (Tb.Sp.) C) µCT analysis of the femur diaphysis from both the 12 and the 28-week experiment. For comparison the control group was normalized to 100%. 1) Cortical Bone Volume (Ct.BV) in % of control, 2) Cortical Thickness (Ct.Th.) in % of control, 3) Cortical Degree of Mineralization of Bone (DMB) in % of control, 4) Endocortical Diameter (Ec.Dm.) in % of control, 5) Endocortical Marrow Volume (Ec.M.V.) in % of control, 6) Periosteal Diameter (P.Dm.) in % of control. µCT was conducted on all bones from mice having completed the study (see methods section).

In alignment with attenuation of bone resorption in the *oc/oc* group the bone volume fraction (BV/TV) of the trabecular compartment of vertebrae was increased by 80%, and the trabecular thickness (Tb.Th.) by 50% at the 12 week time point, while no change in the mineralisation degree (DMB) was observed, when comparing to controls (Figure 3A). In the 28 week experiment, the increases in BV/TV and Tb.Th. in the *oc/oc* group were of the same magnitude as in the 12-week experiment. With respect to DMB a 5% increase was seen in vertebrae of the *oc/oc* compared to the control group after 28 weeks of transplantation. These data were supported by bone histomorphometry on vertebrae showing increased bone volume, as BV/TV, Tb.Th and Tb.N all were increased, while Tb.Sp. was decreased in the *oc/oc* group compared to the control group at the 12-week time point (Figure 3B).

In the femoral cortex, an increase in bone volume (BV) of 12% and cortical thickness of 15% was observed when comparing *oc/oc* to control at 12 weeks, while after 28 weeks the increases were 25 and 30%, respectively (Figure 3C). DMB of the femoral cortex showed a trend towards an increase, but this was not significant. Finally, at the 28-week time point both endocortical diameter and marrow volume were significantly reduced in the *oc/oc* group compared to control, while no changes were seen at the 12-week time point. No changes in periosteal parameters were observed at any of the time points.

### Biochemical markers of bone turnover

Serum samples were collected throughout both experiments to investigate bone turnover markers. To combine the experiments, and to focus on between-group differences, rather than aspects of age, the levels of all markers were normalized to 100% at all time points in the control groups.

**Figure 4:**
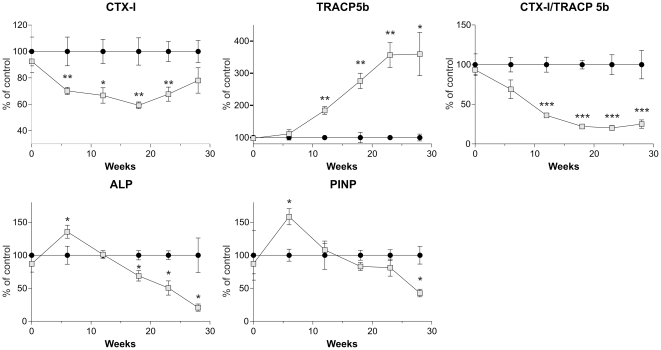
Biochemical markers of bone turnover. Serum samples were collected in both experiments and CTX-I (A), TRACP 5b (B), CTX/TRACP 5b (C), ALP (D), P1NP (E) were measured at baseline and at week 6, 10, 18, 22 and 28, post transplantation. The oc/oc data (gray squares) are plotted as percent of control (black circles) at all time points, and when samples from both experiments were present they were pooled after normalization. The biomarker measurements were conducted in samples from all mice, and for the samples collected during the first 12 weeks on pooled data from both experiments as described in the Methods section.

As seen in Figure 4A the level of the bone resorption marker CTX-I is significantly lower in the *oc/oc* group compared to the control group at all time points, except week 28, where overall CTX-I levels are low due to the advanced age of the mice (baseline CTX-I 50.1±7.9 ng/mL, week 28 CTX-I 24.5±3.5 ng/mL). The marker of osteoclast number TRACP 5b [38]; [39], was highly elevated in the *oc/oc* group from week 12 and throughout, compared with the control group, indicating increased osteoclast numbers *in vivo* (Figure 4B), and the ratio between CTX-I and TRACP 5b, which is used as a index for resorption per osteoclast [38], is markedly lower in the oc/oc group than the control, further confirming that activity per osteoclast is strongly reduced (Figure 4C). Interestingly, the bone formation markers PINP and ALP showed increased levels in the *oc/oc* group compared to the control group at week 6, while the levels returned to normal at week 12, and at the later time points were lower in the *oc/oc* group than the control group (Figure 4D&E). Finally, CTX-II levels, which are indicative of cartilage degradation, were similar in both groups (data not shown).

### Histomorphometric analysis

**Figure 5:**
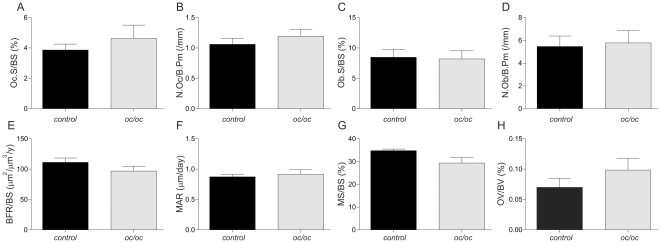
Bone histomorphometry. At termination of the 12-week experiment vertebrae were collected for bone histomorphometry. No significant differences were observed in osteoclast surface per unit bone surface (Oc.S/BS), number of osteoclasts per unit bone perimeter (N.Oc.Pm), osteoblast surface per unit bone surface (Ob.S/BS), number of osteoblasts per unit bone perimeter (N.Ob.Pm), bone formation rate (BFR/BV), mineral appositional rate (MAR), mineralizing surface (MS/BS) or osteoid volume (OV/BV). Bone histomorphometry was conducted on all specimens from the 12-week experiment (see Methods section).

Assessment of osteoclast and osteoblast numbers did not show any differences between the two groups (Figure 5A–D) at the 12-week time point. Furthermore, no differences in the dynamic parameters of bone formation, BRF/BS, MAR, MS/BS, and in osteoid volume (OV/BV) between groups were observed (Figure 5E-H) in the 12-week experiment.

### Bone strength parameters

**Figure 6:**
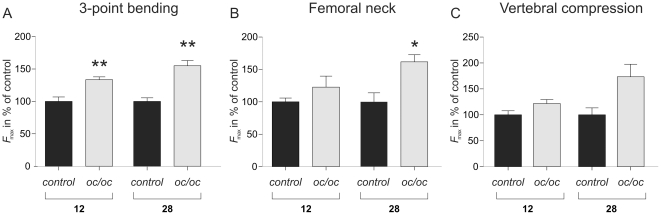
Bone strength analysis. Maximal force achieved at failure (*F*_max_) as determined by 3-point bending test of the femoral cortex (A) or femoral neck (B). In C *F*_max_ was determined by vertebral compression. Bone strength testing was conducted on all bone specimens collected as described in the methods section.

As osteopetrosis is associated with poor bone quality and fractures, we investigated the consequences of induction of osteopetrosis in aged animals using mechanical testing. As for earlier data the values in the control group at both time points were normalized to 100% for comparative purposes. The 3-point bending test of the femoral mid-diaphysis showed a 33% increase in *F*_max_ when comparing *oc/oc* to control at the 12-week time point, while at the 28-week time point the difference was 55% (Figure 6A). At the femoral neck a significant increase of 60% in the *oc/oc* compared to control was seen at the 28-week time point, while at the 12-week time point a trend towards increased strength was seen (Figure 6B). In the vertebrae, no significant differences were observed, although the trends followed the other mechanical tests (Figure 6C).

### Assessment of bone structure

**Figure 7:**
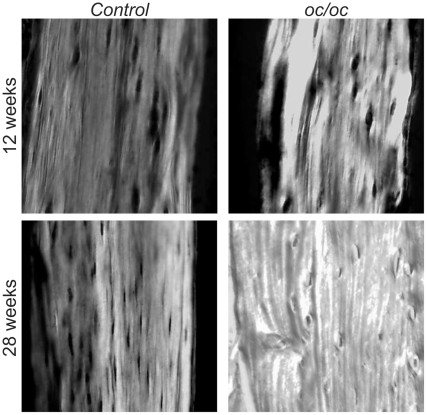
Analysis of bone structure. Bone structure was assessed using polarized light microscopy as described in the methods section.

To further understand the effects of transplantation with the *oc/oc* cells, bone structure was analyzed using polarized light microscopy. In figure 7 it is clearly shown that cortical bone is organized in well-structured lamellae indicating that transplantation has no detrimental effect on bone structure. Similar findings were obtained in trabecular bone (data not shown).

## Discussion

The hematopoietic nature of osteopetrosis was established in the mid 1970s by transplantations of spleen cells from either healthy donor mice to osteopetrotic mice, or *vice versa* [40], [ 41], [ 41, 42]. Transfer of *oc/oc* splenocytes into healthy young mice led to increased bone weight [30], however other bone parameters were not examined.

Here we present novel data on the establishment of osteopetrosis in skeletally mature mice, in order to isolate the effect of non-resorbing osteoclasts on mature bone from the influence of non-resorbing osteoclasts on skeletal development and the resorption of mineralized cartilage in young mice.

Using fetal liver cells as a source of hematopoietic cells [27] an engraftment level in excess of 95% was obtained at 12 weeks, and the levels were around 98% 28 weeks after transplantation, confirming transplantation efficiency. No signs of hepatosplenomegaly were observed in any of the experiments, and no alterations in the cells of the hematopoietic lineages were observed, in contrast to haemopoietic defects observed in mice with lifelong osteopetrosis [10], [ 26], [ 36], [ 37]. This, not surprisingly, indicates that the haemopoietic phenotype of *oc/oc* mice is a developmental phenotype, in which the anemia effect is compounded by the complete lack of bone marrow cavities in mice with osteopetrosis due to defective acid secretion [10], [ 26], [ 36], [ 37]. These findings are further supported by studies in RANKL and RANK deficient mice, which have a less severe bone phenotype than *oc/oc*, *Atp6i* and ClC-7 deficient mice, and accordingly have only mild changes in the hematopoietic system and show no sign of anemia [43], [ 44]; however, to fully understand these differences more detailed analyses are needed.

To validate that the osteoclasts were non-resorbing, osteoclastogenesis and bone resorption were evaluated using spleen and bone marrow-derived osteoclasts from mice transplanted with either *oc/oc* or control hematopoietic cells. These data confirmed functional deficiency of the *oc/oc* osteoclasts, while showing no changes in osteoclastogenesis, as expected from a previous study of osteoclasts lacking the a3 subunit of the proton pump [37], as well as studies of osteoclasts with defective acid secretion [10], [ 11], [ 24], [ 37], [ 45]. These data also fit well with earlier findings showing that the increased numbers of osteoclasts in the acid secretion deficient mice are caused by increased survival of the osteoclasts, but not by changes in osteoclastogenesis [11], [ 46], [ 47].

In both human and murine osteopetrosis forms caused by defective acid secretion by the osteoclasts, bone quality is low and fractures are frequent [48]-[50]; however the explanation for this has never been clear, and the possibilities include over-suppression of bone turnover, accelerated osteoblast function, the presence of woven, and therefore immature, bone, and finally failure to resorb calcified cartilage [9], [ 21]-[23].

Our mechanical testing data of both trabecular and cortical bone indicate that induction of osteopetrosis in adult animals leads to increased bone strength. Since we found almost no remaining calcified cartilage, as well as no changes in cartilage degradation markers, these data suggest that it is the remaining calcified cartilage in the bones of young osteopetrotic mice that is the basis of the poor bone strength [51]. However, the gained bone was notably devoid of woven bone, a phenomenon observed in classical osteopetrosis, and thus the increase in lamellar bone volume is likely to also contribute the increased bone strength observed in the adult osteopetrotic mice. The tests performed do not take into account whether the bones from the transplanted osteopetrotic mice are more brittle at the tissue level; however, as the degree of mineralization only increases modestly and more slowly than breaking strength, this does not appear to be the cause. Furthermore, the normal bone structure observed in the *oc/oc* groups also supports the notion that the gained bone is normal at all levels. Importantly, these experiments do not take into account whether the poor bone quality observed in young *oc/oc* mice is due to expression of the a3 subunit of the V-ATPase in non-hematopoietic cells, i.e. gastric the parietal cells which are involved in calcium homeostasis [52]; however, as the fragility of osteopetrotic bone is common to multiple types of osteopetrosis this does not appear to be likely. Increased bone strength has been observed in cortical, but not vertebral bone, of cathepsin K deficient mice [53], and in cortical bone of Ae2_a,b_ deficient mice [54]. However, these mice also have thickened cortices, as opposed to acid secretion deficient mice, which have very little if any normal cortex [25]. Furthermore, the Ae2_a,b_ and cathepsin K deficient mice also show less dramatic accumulation of calcified cartilage in the bone marrow cavities [54]; [55]. CT analysis of the bones showed increased bone volume in both trabecular and cortical compartments. Interestingly, the increase in bone volume in the vertebrae appeared to plateau after only three months, while the increase in femoral bone volume was continuous. Furthermore, the increase in cortical bone volume appeared to be mainly caused by a reduction in endocortical resorption, as endocortical diameter was reduced, but periosteal parameters were not changed.

The increase in bone volume is explained by the changes observed in biochemical markers. Bone resorption CTX-I was significantly reduced, which is as expected from the *in vitro* data, and this reduction in bone resorption most likely explains most of the increase in bone volume and bone strength. This differs from data presented in osteopetrosis models where the defect is present during bone development [24], [ 56]. However, a study conducted in ClC-7 deficient mice, which have a phenotype closely matching that of the *oc/oc* mice, indicated that the high resorption marker levels originate from resorption of non-mineralized matrices, which have not been removed correctly during endochondral ossification. The reasoning being that CTX-I release occurred completely independent of acid secretion by the osteoclast, and thus independent of resorption of calcified bone [24], [ 25].

As expected from previous studies, osteoclasts numbers increase with defective acid secretion [17], [ 18], [ 20], [ 46], [ 47], [ 57], [ 58]. In confirmation of a large reduction in resorptive capacity per osteoclast, the CTX-I/TRACP5b ratio was suppressed strongly [38]. The bone formation markers PINP and ALP were both increased by 6 weeks after transplantation, by 12 weeks they had returned to control levels, and at the later stage both these markers were decreased. The effect of this transient increase in bone formation on bone volume and strength is not clear, but the lower level of bone formation after 12 weeks may explain why the vertebral bone volume plateaus from that time, despite the ongoing reduction in resorption.

Taken together, the biochemical markers show that in early stages of induced osteopetrosis, bone formation is uncoupled from bone resorption, corresponding well to previous data from osteoclast-rich forms of osteopetrosis caused by defective acid secretion [17], [ 19], [ 20]. In contrast, in osteoclast-poor forms of osteopetrosis bone formation is low from the starting point [59], [ 60], and in bisphosphonate or OPG-treated animals bone formation levels decrease rapidly after onset of treatment [61].

With respect to histomorphometry, we could neither confirm an increase in osteoclast numbers, nor a change in bone formation at week 12; and we speculate that it may require more time to see these differences by histomorphometry, as the early effects are mainly driven by the reduction in resorption, while the increased osteoclast survival is not seen until week 12 and at this time point the effect on the osteoclast marker TRACP 5b is not very dramatic. Furthermore, the biomarkers reflect the whole skeleton, whereas histomorphometry reflects only the vertebrae, and thus the markers will accumulate systemic changes. These biomarkers have, on the other hand, been shown to clearly reflect larger changes observed by histomorphometrical analysis [38], [ 61], [ 62].

Although bone formation decreases at later stages, these data indicate that when acid secretion by the osteoclast is attenuated a period of anabolic activity occurs. However, the duration and extent of this activity will need further investigation as osteoclast-rich osteopetrosis patients appear to have normal or increased levels of bone formation, even though bone resorption per osteoclast is significantly reduced [17]–[20].

The mechanisms controlling the coupling of bone formation to bone resorption have long been under debate, and several recent lines of evidence have indicated that the osteoclasts themselves, rather than their activity, are essential for the control of bone formation [2], [ 4], [ 17]–[20], [ 59], [ 60], [ 63]–[67]. In addition to the acid secretion deficient mice and patients, studies in cathepsin K deficient mice, and cathepsin K inhibitors in monkeys, have shown increased bone formation, despite reduced bone resorption, although the effects appear to be bone type dependent [53], [ 68], [ 69]. One study showed that inhibition of cathepsin K in osteoclasts *in vitro* led to augmented release of anabolic factors from the resorption compartment, while inhibition of acid secretion by bafilomycin prevented the release of anabolic factors [70]. All these data strongly indicate that the osteoclasts possess the ability to induce an anabolic response in osteoblasts. In addition, evidence has been provided that osteoclast-derived ephrinB2 might promote bone formation by acting upon receptor EphB4 in the osteoblast lineage, by a contact-dependent mechanism [71]. However, whether these are the factors involved in the uncoupling seen in these mice, and to what extent the coupling molecules originate from either bone resorption or directly from the osteoclasts, remain to be studied.

In conclusion, we here show an increase in bone volume and bone strength when osteopetrosis due to impaired acid seretion from osteoclasts is induced in adult mice. This suggests that the low bone quality seen in osteopetrosis in young animals most likely is due to the developmental nature of the phenotype. Furthermore, these data support that an “uncoupling” between bone resorption and bone formation can be obtained when attenuating acid secretion by the osteoclasts. Finally, the substantial increase in bone volume and bone strength observed in otherwise healthy mice with attenuated osteoclast acidification warrant further investigation of the osteoclastic V-ATPase as a therapeutic target for osteoporosis.

## Supporting Information

Figure S1 **Flow cytometry analysis of the major hematopoietic cell lines conducted using antibodies against B220, CD3, Mac1 and Gr1 showing no significant differences in the percentages of these cells.**
(TIF)
