# Supplementary figures and images for: Dissociation of Bone Resorption and Bone Formation in Adult Mice with a Non-Functional V-ATPase in Osteoclasts Leads to Increased Bone Strength
Source: PLoS One. 2011 Nov 7;6(11):e27482. doi: 10.1371/journal.pone.0027482 (PMC3210177; doi:10.1371/journal.pone.0027482)

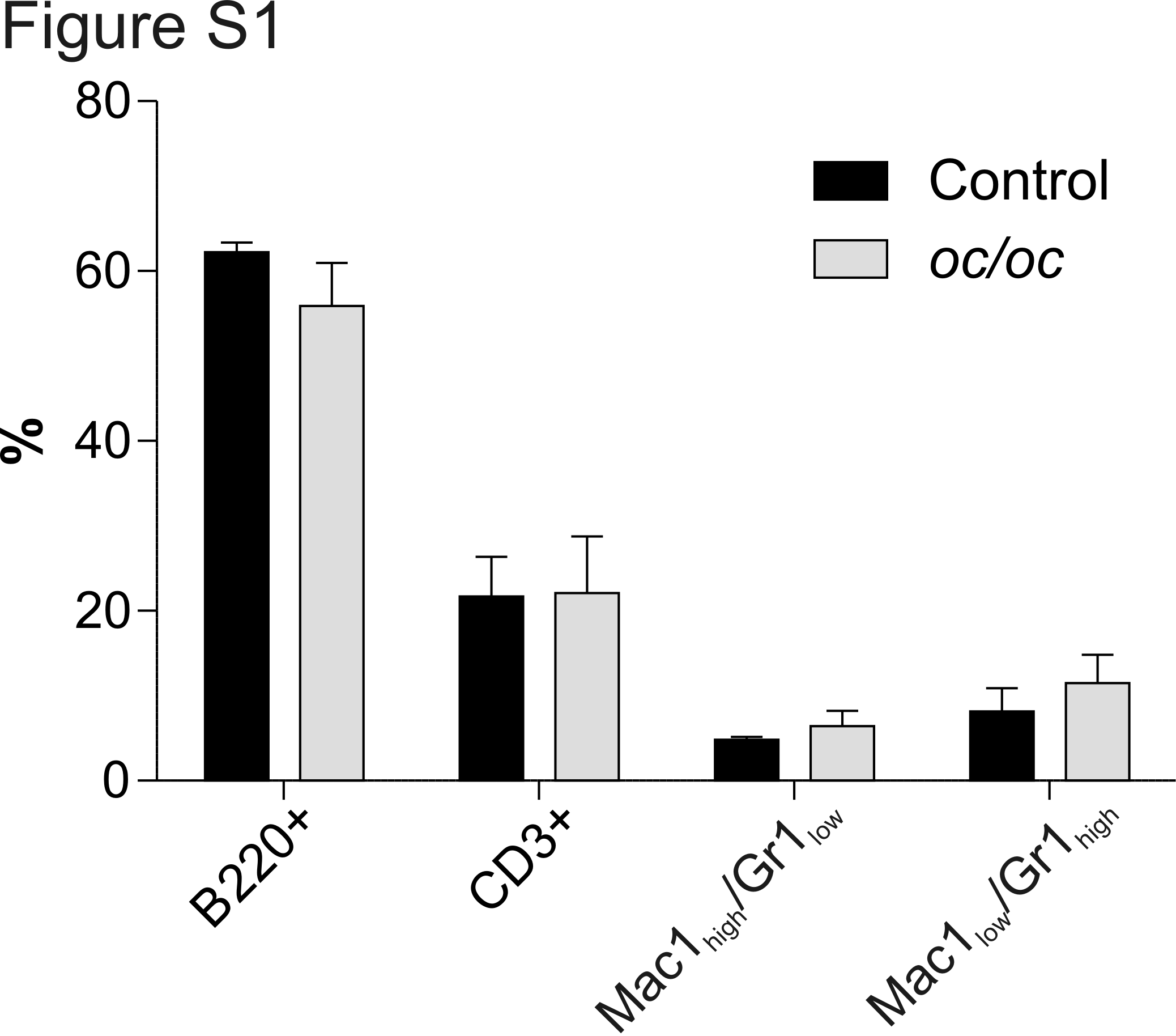

Supplement: Figure S1 — Flow cytometry analysis of the major hematopoietic cell lines conducted using antibodies against B220, CD3, Mac1 and Gr1 showing no significant differences in the percentages of these cells. (TIF) [file pone.0027482.s001.tif]
